# Sudden Death after Parturition, with Air in the Veins

**Published:** 1857-11

**Authors:** George May


*Sudden Death after Parturition, with Air in the Veins. By Geo. May, Jr., Esq.*

That death may result from the entrance of air into the veins during surgical operations, has long been known to the profession; but that it might be a cause of danger after parturition (as suggested by Legallois in 1829), did not obtain the notice it deserved, until Dr. Cormack read a paper on the subject before the Westminster Medical Society in 1850. I propose to allude briefly to the cases narrated by Dr. Cormack, and then to give the details of three that have occurred in this neighborhood.

In 1841, Dr. Bessems attended a labour, in which there was hemorrhage with retention of placenta. On the fourth day after her confinement, whilst an injection was being thrown into the uterus, she suddenly exclaimed that she was suffocated, and died in three minutes. Air was found in the heart and veins.

M. Lionet, of Corbeil, attended a lady, aged 27. She was much frightened during the last month of pregnancy, and did not completely recover her strength; but her labour was natural, and not attended with hemorrhage. She soon, however, became faint, breathed with difficulty, and expired five hours after delivery. Air was found in the heart and in the cerebral veins.

Dr. Wintrich, in 1848, published a case of rapid death after parturition. Convulsive movements and suffocation followed the expulsion of the infant and partial separation of the placenta. Air was found in the venous system.

Professor Simpson mentions a case in which death occurred a few hours after a delivery, accompanied with hemorrhage and alternate contractions and relaxations of the uterus. Air was found to have entered through the uterine veins.

Dr Lever mentions three cases; in all of them there was hemorrhage, and death a few hours after labour. Air was found in the uterine and other veins.

In 1850, Mr. Berry, of Birmingham, attended a primipara, aged 22. There was little hemorrhage, and she appeared to be going on well for six hours; she then became affected with difficulty of breathing and faintness, and expired in less than an hour. Air was found in the heart. The uterine veins were patulous.

Case 1.—The case of which an abstract is here given, was read before the Reading Pathological Society, by Mr. Taylor, of Wargrave.

In September, 1841, Mrs. ——, aged 30, was taken in labour with her third child. The labour progressed naturally; but no urine having been passed, Mr. Taylor was in the act of introducing a catheter, when a sever pain occurred. The liquor amnii was discharged to the amount of three-fourths of a pint. The woman suddenly exclaimed, "Oh! how faint I feel," was convulsed for a moment, and then expired. By the last pain, the head had been forced partly from the outlet. An attempt was made to remove the child without success.

A *post-mortem* examination was made forty-eight hours after death. The uterus extended above the umbilicus; no portion was separated. A few days before her labour, she had a copious discharge of blood. There was little blood in the uterus. The bladder was empty. The lower vena cava was empty. The heart was healthy. The right auricle was thin, almost transparent, and distended with air. Hardly a trace of blood existed in the heart. The brain and membranes were healthy. In the spine, between the theca and the cord, there was considerable effusion of fluid blood, but none within the sheath.

CASE 2.—I am indebted to Mr Smith, of Whichurch, for the details of the following case:

Mrs. T., between 38 and 40 years of age, was confined of her sixth child, a male, on the morning of May 7, 1852, about 8 a. m., and her attendant left her shortly afterwards, as he said, very comfortable. As, however, she had severe after-pains, an opiate was sent for. Mr. Smith was summoned to her about 2 p. m., and on his arrival, he found she had just died. She complained of excessively severe after-pains, together with great oppression about the chest, and feelings of sinking and exhaustion and extreme restlessness. In answer to inquiries as to whether there had been any hemorrhage, the attendants stated that there had not.

A *post-mortem* examination was performed the same evening, the body not being quite cold. The abdominal viscera were all free from disease. On opening the uterus, which was large, there was found a considerable quantity of coagulated blood; but not by any means enough to satisfy one that loss of blood was the cause of death. The uterus contained also a considerable piece of the placenta adhering to its internal surface. In the chest were old adhesions between the pleura costalis and pulmonalis. The heart appeared distended; not that it was enlarged, properly so called, but that it had an appearance of distension, which was evidently on the right side of the organ. On opening the right auricle, a quantity of air escaped with a sort of a little puff, and the organ was at once reduced to its proper dimensions. No disease was found in its substance or valves. The left ventricle contained a small clot.

CASE 3.—In the autumn of 1855, Mrs. E., aged 28, was delivered of her third child, after a natural labour. She had become sufficiently convalescent to resume her household duties; but on the eighth day, she was taken suddenly ill, and expired before Mr. Walford arrived.

I assisted at the *post-mortem* examination the following day. No unusual appearance was observed, until the liver was sliced; it was then noticed that frothy blood escaped, and, a further examination being made, air was discovered in the vena cava inferior and vena portæ; and the right side of the heart was distended with frothy blood. The uterus was of its usual size for the eighth day. There was no sign of decomposition about the body.

*Remarks.*—It does not appear to be generally admitted that the entrance of air through the uterine sinuses can cause death; but if we recollect that Dr. Cless, of Stuttgardt, examined the bodies of 1,200 patients, who had died of various diseases, without finding air in the heart; that in the eleven cases here alluded to, death was more or less sudden, and could be explained by no *post-mortem* appearances; that the development of gas from putrefaction was quite out of the question, some of the bodies being warm at the time of examination; and that these cases present an analogy with those in which air enters the veins during operations and experiments; I think we are forced to the conclusion that the entrance of air through the uterine veins was the cause of death.

I will, however, shortly send some examples to prove that the local generation of air may in some cases prove fatal.—*British Med. Journal*, June 6, 1857.

